# Biophysical analysis of the effect of chemical modification by 4-oxononenal on the structure, stability, and function of binding immunoglobulin protein (BiP)

**DOI:** 10.1371/journal.pone.0183975

**Published:** 2017-09-08

**Authors:** Dinen D. Shah, Surinder M. Singh, Monika Dzieciatkowska, Krishna M. G. Mallela

**Affiliations:** 1 Department of Pharmaceutical Sciences, Skaggs School of Pharmacy and Pharmaceutical Sciences, University of Colorado Anschutz Medical Campus, Aurora, Colorado, United States of America; 2 Biological Mass Spectrometry Facility, School of Medicine, University of Colorado Anschutz Medical Campus, Aurora, Colorado, United States of America; 3 Program in Structural Biology and Biochemistry, University of Colorado Anschutz Medical Campus, Aurora, Colorado, United States of America; Southern Illinois University School of Medicine, UNITED STATES

## Abstract

Binding immunoglobulin protein (BiP) is a molecular chaperone important for the folding of numerous proteins, which include millions of immunoglobulins in human body. It also plays a key role in the unfolded protein response (UPR) in the endoplasmic reticulum. Free radical generation is a common phenomenon that occurs in cells under healthy as well as under stress conditions such as ageing, inflammation, alcohol consumption, and smoking. These free radicals attack the cell membranes and generate highly reactive lipid peroxidation products such as 4-oxononenal (4-ONE). BiP is a key protein that is modified by 4-ONE. In this study, we probed how such chemical modification affects the biophysical properties of BiP. Upon modification, BiP shows significant tertiary structural changes with no changes in its secondary structure. The protein loses its thermodynamic stability, particularly, that of the nucleotide binding domain (NBD) where ATP binds. In terms of function, the modified BiP completely loses its ATPase activity with decreased ATP binding affinity. However, modified BiP retains its immunoglobulin binding function and its chaperone activity of suppressing non-specific protein aggregation. These results indicate that 4-ONE modification can significantly affect the structure-function of key proteins such as BiP involved in cellular pathways, and provide a molecular basis for how chemical modifications can result in the failure of quality control mechanisms inside the cell.

## Introduction

A newly synthesized protein in cell encounters an environment of molecular crowding with a high concentration of other proteins, macromolecules, and various cellular components [1]. The role of a chaperone protein is to guide and assist the synthesized protein in acquiring its native conformation. Chaperone proteins help their substrate proteins to fold correctly, and prevent misfolding and aggregation. In addition, chaperones play crucial roles during perturbations of endoplasmic reticulum (ER) homeostasis, which causes accumulation of unfolded or aggregated proteins [2]. These perturbations can be because of various factors that include stress, disease states, inflammation, alcohol, and smoking, which initiates unfolded protein response (UPR). UPR involves upregulating chaperone proteins such as binding immunoglobulin protein (BiP) to help the cell survive the stress [3]. Thus, chaperones play the role of quality control in the cell and are vital for its proper functioning.

Numerous secretory as well as membrane proteins undergo folding and post-translational modifications in ER. This activity is mainly regulated by ATP-dependent chaperone proteins [4,5]. The concentration of proteins in ER often reaches 100 mg/ml [6]. At such high concentrations, it is imperative for the cell machinery to have effective chaperones that will control and regulate protein folding and aggregation [7]. One such important chaperone protein is BiP (also known as glucose-regulated protein GRP78). BiP is a key member of the Hsp70 family and is the only Hsp70 found in the ER. BiP is a critical component of the UPR, and is the first chaperone protein that has been shown to bind immunoglobulin molecules that were incompletely assembled, thus preventing their transport from ER [8–10]. Almost one third of the proteins in the cell are targeted to ER before they are trafficked to their cellular locations [11]. BiP is responsible for the correct folding and preventing aggregation of these proteins which include millions of antibodies. In addition, BiP is critical for embryonic development; its absence has been shown to be lethal using an embryonic mice model [12]. BiP also plays a key role in many disease states. Because of its chaperone activity, BiP is overexpressed in tumor cells, and these cells use the pro-survival tendencies of BiP to avoid apoptosis. Hence, controlling BiP expression in tumor cells has been a promising strategy for developing anti-cancer treatments [13,14]. In addition, failure of BiP and HSP chaperone systems has been implicated in several neurodegenerative disorders, because most neurological disorders are a result of the accumulation and aggregation of misfolded proteins [15,16]. In addition, modified expression of BiP has been shown to be a critical factor in other diseases such as diabetes [12,14–20]. Hence, the loss of BiP function could amount to a potential cascade effect that could affect the cellular function. Therefore, understanding the factors that trigger the loss of the stability and function of BiP is important in understanding various associated disorders.

Generation of excess free radicals and their reactive byproducts is a major cause of the loss of cellular function in response to various factors, such as stress, ageing, pollution, smoking, and alcohol. Correspondingly, free radicals have been shown to trigger various diseases [21–23]. Free radicals attack biomolecules including lipids that are main constituents of the cell membranes, and generate reactive lipid peroxidation products such as 4-oxynonenal (4-ONE) [24–26]. Polyunsaturated fatty acids like arachidonic acid and linoleic acid generate a significant amount of these reactive aldehydes by autoxidation [27]. Cellular concentration of 4-ONE in response to alcohol and smoking has been shown to reach up to 10 mM [26]. These highly reactive lipid peroxidation products attack other macromolecules such as proteins in the cell. Earlier proteomic analysis indicated that BiP is a key protein that is modified by 4-ONE [28]. In this manuscript, we examined how this chemical modification affects the structure, stability, and function of BiP, which might lead to a mechanistic understanding of the ER homeostasis failure in response to external factors such as alcohol and smoking.

**Fig 1:**
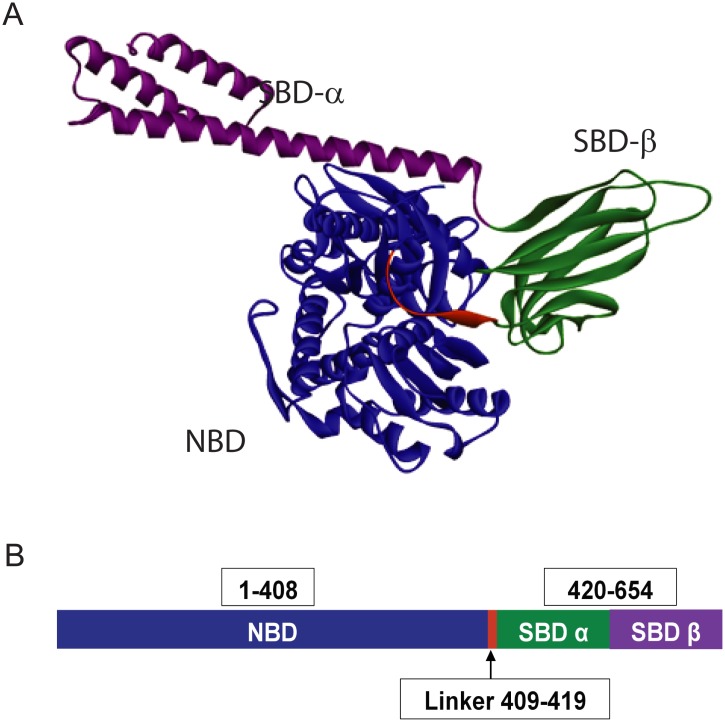
(A) Molecular Structure of BiP (Protein Data Bank ID: 5E84). (B) Domain architecture of BiP. Nucleotide binding domain (NBD) is shown in blue color, whereas the SBD-α and SBD-β are shown in green and purple colors, respectively. Hydrophobic linker connecting the NBD and SBD is shown in red color.

Recently solved crystal structure of BiP [29] is shown in Fig 1. It contains two domains, a nucleotide binding domain (NBD) and a substrate binding domain (SBD). ATP binds to BiP at its NBD. SBD has two subdomains: SBD-βand SBD-α. SBD-β is predominantly of β-sheet structure, whereas SBD-α is predominantly α-helical. Immunoglobulins bind at SBD-β, whereas SBD-α acts as a lid controlling the interactions between NBD and SBD [30]. The binding of ATP to NBD causes structural perturbations in the protein, which facilitates the binding of immunoglobulins to the SBD. Subsequent hydrolysis of ATP causes BiP to release the substrate [31]. ATPase activity has been shown to be necessary for the correct functioning of HSP70 chaperone protein family of which BiP is a part of [32]. BiP mutants with faulty ATPase activity have been shown to be incapable of releasing immunoglobulin light and heavy chains [33]. In this manuscript, we probed the effect of chemical modifications on the function of BiP, and examined whether such functional changes could be explained in terms of changes in protein structure and stability.

## Materials and methods

### Materials

BiP plasmid was obtained from Harvard Plasmid Bank (https://plasmid.med.harvard.edu/PLASMID/Home.xhtml). We used a modified pET-28a vector, pET-SUMO (a generous gift from Christopher Lima, Sloan-Kettering Institute) as the expression vector for BiP. 4-oxo-2-nonenal (4-ONE) was purchased from Cayman chemicals (Cat# 10185), Michigan, USA. Porcine citrate synthase (Cat# C3260-1KU) and malachite green oxalate salt (Cat# M9015) were bought from Sigma Aldrich, St Louis, MO. Lucifer yellow iodoacetamide dye (Cat# 6592) was purchased from Setareh Biotech, Eugene, OR, USA. Immunoglobulin peptide HTFPAVLGSC was purchased from Biomatik, Delaware, USA.

### BIP expression and purification

The BiP gene was first cloned into the pET-SUMO vector using PCR. The gene was amplified using DH5α *Escherichia coli* cell line by heat shock treatment. The sequence of BiP was confirmed using DNA sequencing. The plasmid was then transferred to BL21 (DE3) *Escherichia coli* cells using PCR and heat shock treatment. Protein expression was induced by 0.4 mM isopropyl β-D-1-thiogalactopyranoside (IPTG) for 16 hours at 15°C and purified using Ni-sepharose column. The His-SUMO tag was cleaved with Ulp1 protease and the purified protein was dialyzed in PBS buffer (100 mM Na phosphate, 150 mM NaCl, pH 7).

### BIP modification with 4-ONE

The protein (10 μM) was modified with 4-ONE (10x, 50x, and 100x) by overnight incubation at room temperature. Following modification, sodium borohydride was used as a reducing agent to stabilize the protein adducts by incubating for another 4 hrs.

### Circular dichroism (CD)

The Chirascan plus spectrophotometer (Applied Photophysics) was used for the CD measurements. The CD scans for both wild-type and modified BiP were recorded at 0.4 μM protein concentration in PBS and the signals were plotted as mean residue ellipticity (MRE). MRE was calculated from the measured CD values in millidegrees using the equation,

[θ]=millidegrees/ (pathlength in millimeters × molar concentration of protein × number of residues) (1)

### Fluorescence

PTI Quantamaster fluorimeter was used for measuring the fluorescence spectra of wild-type and modified BiP. Protein concentration of 0.5 μM was used for these measurements. The excitation wavelength was 280 nm.

### Denaturant melts

Denaturant melts were carried out on both wild-type and modified proteins at a protein concentration of 0.5 μM and with urea concentrations ranging from 0 M to 9 M urea. The urea concentration was calculated using changes in refractive index of the solution [34]. Refractive index was measured using the Bausch and Lomb Abbe-3L refractometer. The CD signal was monitored at 222 nm. For fluorescence measurements, the excitation wavelength was 280 nm and the emission wavelength was 364 nm as it was the wavelength that showed maximum difference between the native and denatured proteins. Protein-urea solutions were equilibrated for 2 hours prior to CD and fluorescence measurements.

### Thermal melts

For wild-type and modified BiP (1 μM each in PBS buffer), changes in the far-UV CD signal at 222 nm were monitored. Protein fluorescence with excitation wavelength at 280 nm and emission wavelength at its λ_max_ = 330 nm was monitored. Both the thermal denaturation melts were performed as a function of increasing temperature at a scan rate of 1°C/min.

### Differential scanning calorimetry (DSC)

Microcal VP DSC was used to determine the effect of chemical modification on the unfolding of individual domains (NBD and SBD) of BiP. For these measurements, proteins were used at a concentration of 13 μM. The DSC scans were done from 25°C to 90°C at a scan rate of 1°C/ min.

### Malachite green assay

ATPase activity of BiP was measured using the malachite green-ammonium molybdate assay. The free phosphate generated from ATP hydrolysis was detected as a change in the absorbance of malachite green-phosphomolybdate-triethanolamine dye complex at 660 nm. A standard curve was generated with increasing concentrations of free phosphate against absorbance values. ATP hydrolysis in the absence of BiP was measured as a control to exclude false positives. The protein concentration used was 0.75 μM BiP in triethanolamine (TEA) buffer. ATP concentration was 1 mM.

### Nucleotide binding assay

Binding of ATP to BiP before and after chemical modification was monitored using near-UV CD on a Chirascan Plus Applied Photophysics instrument. The change in the tertiary structure of the protein because of ATP binding was followed using near-UV CD. Binding of ATP caused a distinct change in the CD spectra with a maximal difference at 266 nm. Concentration of BiP used was 15 μM. ATP in buffer was used as blank for each measurement. Changes in the near-UV CD signal were plotted against increasing ATP concentration. The dissociation constant (K_d_) values were determined by fitting the binding curves to a single site ligand binding model using the following formalism.

If P represents BiP and L represents ATP, the measured signal (S) can be written as

$$S=\frac{S_{P}\left[P\right]+S_{PL}\left[PL\right]}{\left[P\right]+\left[PL\right]}$$ (2)

where S_P_ and S_PL_ represent the signals corresponding to the free BiP and BiP bound to ATP, respectively. The above equation simplifies to

$$S=S_{P}+\frac{\left(S_{PL}-S_{P}\right)\left[PL\right]}{{\left[P\right]}_{T}}$$ (3)

where [P]_T_ is the total protein concentration used, which is the sum of free protein concentration [P] and the bound protein concentration [PL].

The concentration of PL can be obtained from the expression for K_d_ as

$$K_{d}=\frac{\left[P\right]\left[L\right]}{\left[PL\right]}$$ (4)

which simplifies to a quadratic equation

$${\left[PL\right]}^{2}-\left({\left[P\right]}_{T}+{\left[L\right]}_{T}+K_{d}\right)\left[PL\right]+{\left[P\right]}_{T}{\left[L\right]}_{T}=0$$ (5)

where [L]_T_ is the total ATP concentration used in the experiment. Solving the above quadratic equation leads to a root

$$\left[PL\right]=\frac{\left({\left[P\right]}_{T}+{\left[L\right]}_{T}+K_{d}\right)-\sqrt{{\left({\left[P\right]}_{T}+{\left[L\right]}_{T}+K_{d}\right)}^{2}-4{\left[P\right]}_{T}{\left[L\right]}_{T}}}{2}$$ (6)

By substituting this value of [PL] into the above Eq 3, we determined the K_d_ values for ATP binding to the wild-type and modified BiP.

### Citrate synthase (CS) assay

The non-specific chaperone activity of BiP before and after chemical modification was measured in terms of its ability to rescue CS from aggregation at 43°C. The ratio of CS to BiP was optimized for maximal rescue activity. A ratio of 1:2 (CS (2 μM): BiP (4 μM)) was used. CS was incubated isothermally at 43°C without and with BiP, and its isothermal aggregation was monitored using light scattering at 350 nm using an Agilent 8453E UV-visible spectrometer.

### Peptide binding assay

Binding affinity of BiP to its specific substrate, immunoglobulin, before and after modification was tested using a peptide HTFPAVLGSC derived from the CH1 domain of immunoglobulin. The peptide was labelled with lucifer yellow fluorescent dye using iodoacetamide chemistry. The free dye was separated from the labelled peptide by dialysis using a 1 kDa cutoff. Fluorescence anisotropy of covalently attached lucifer yellow was monitored as a function of increasing concentrations of BiP at a fixed concentration of the labeled peptide (12.5 μM). Lucifer yellow was excited at 430nm and the emission was detected at 482nm.

### Mass spectrometry

The molecular weight of the intact protein was determined using Omniflex MALDI-TOF mass spectrometry. The samples were cleaned using ZipTip. To identify the specific amino acids that were chemically modified by 4-ONE, tryptic digest was done on the protein bands corresponding to the control and modified BiP samples on SDS-PAGE, and the peptide fragments were analyzed by mass spectrometry. Exact protocols are described below.

#### Sample preparation for tryptic digest

The protein band corresponding to the BiP protein from SDS-PAGE was destained in 200 μl of 25 mM ammonium bicarbonate in 50% v/v acetonitrile for 15 min, and then 200 μl of 100% acetonitrile was applied for 15 min at room temperature. Dithiothreitol (DTT) was added to a final concentration of 10 mM and incubated at 65°C for 30 min to reduce the disulfide bonds. Protein cysteines were alkylated with 55 mM iodoacetamide for 30 min at room temperature in the dark. The iodoacetamide was then removed, and washes were performed with 200 μl of distilled water followed by the addition of 100 μl of acetonitrile. Then, acetonitrile was removed, and 50 μl of the 0.01 μg/μl trypsin solution was added to the gel plugs and allowed to rehydrate at 4°C for 30 min. The plugs were then placed at 37°C and allowed to digest overnight. The tryptic mixtures were acidified with formic acid up to a final concentration of 1%. Peptides were extracted three times from the gel plugs using 50% acetonitrile, 1% formic acid, concentrated under vacuum (SpeedVac, Savant ThermoFisher) to approximately ~20 μL, and subjected to LC-MS/MS analysis. If necessary, they were stored at −20°C.

#### LC–MS/MS analysis

Samples were analyzed on an LTQ Orbitrap Velos mass spectrometer (ThermoFisher Scientific) coupled to an Eksigent nanoLC-2D system through a nanoelectrospray LC−MS interface. A volume of 8 μL of sample was injected into a 10 μL loop using the autosampler. To desalt the sample, material was flushed out of the loop and loaded onto a trapping column (ZORBAX 300SB-C18, dimensions 5 x 0.3 mm x 5 μm) and washed with 0.1% formic acid at a flow rate of 5 μL/min for 5 minutes. The analytical column was then switched on-line at 0.6 μl/min over an in house-made 100 μm i.d. × 200 mm fused silica capillary packed with 4 μm 80Å Synergi Hydro C18 resin (Phenomex; Torrance, CA). After 10 min of sample loading, the flow rate was adjusted to 0.35 mL/min, and each sample was run on a 90-min linear gradient of 5–40% acetonitrile with 0.1% formic acid to separate the peptides. LC mobile phase solvents and sample dilutions used 0.1% formic acid in water (Buffer A) and 0.1% formic acid in acetonitrile (Buffer B) (Chromasolv LC–MS grade; Sigma-Aldrich, St. Louis, MO). Data acquisition was performed using the instrument supplied Xcalibur^™^ (version 2.1) software. The mass spectrometer was operated in the positive ion mode. Each survey scan of *m/z* 400–2,000 was followed by collision-assisted dissociation (CAD) MS/MS of twenty most intense precursor ions. Singly charged ions were excluded from CID selection. Normalized collision energies were employed using helium as the collision gas.

#### Database searching, protein identification

MS/MS spectra were extracted from raw data files and converted into mgf files using a PAVA script (UCSF, MSF, San Francisco, CA). These mgf files were then independently searched against human SwissProt database using an in-house Mascot^™^ server (Version 2.2.06, Matrix Science). Mass tolerances were +/- 15ppm for MS peaks, and +/- 0.6 Da for MS/MS fragment ions. Trypsin specificity was used allowing for 1 missed cleavage. Met oxidation, protein N-terminal acetylation, peptide N-terminal pyroglutamic acid formation and lysine, cysteine and histidine 4-ONE modifications were allowed for variable modifications while carbamidomethyl of Cys was set as a fixed modification.

Scaffold (version 4.3.2, Proteome Software, Portland, OR, USA) was used to validate MS/MS based peptide and protein identifications. Peptide identifications were accepted if they could be established at greater than 95% probability as specified by the Peptide Prophet algorithm. Protein identifications were accepted if they could be established at greater than 99.0% probability and contained at least two identified unique peptides.

## Results

### Modification of BiP by 4-ONE induced the formation of high molecular weight, soluble BiP aggregates

**Fig 2:**
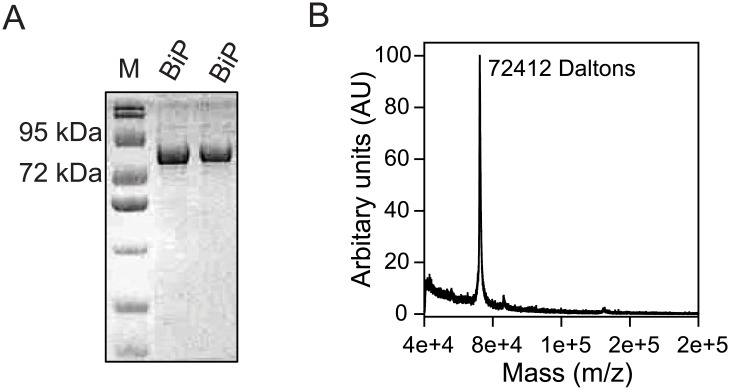
Expression and purification of BiP. (A) SDS-PAGE of purified BiP. Lane labeled M contained the molecular weight markers. (B) Mass spectrometry of intact full length BiP confirmed the purity of the protein.

BiP was expressed and purified to high homogeneity. SDS-PAGE indicated a single species with a molecular weight expected for BiP (Fig 2A). Although it is of 72 kDa, BiP has been shown earlier to run with an apparent molecular weight close to 78 kDa [35]. In addition, mass spectrometry of the intact, full-length BiP showed a single species with a molecular weight of 72 kDa (Fig 2B), close to the calculated value from its amino acid sequence.

**Fig 3:**
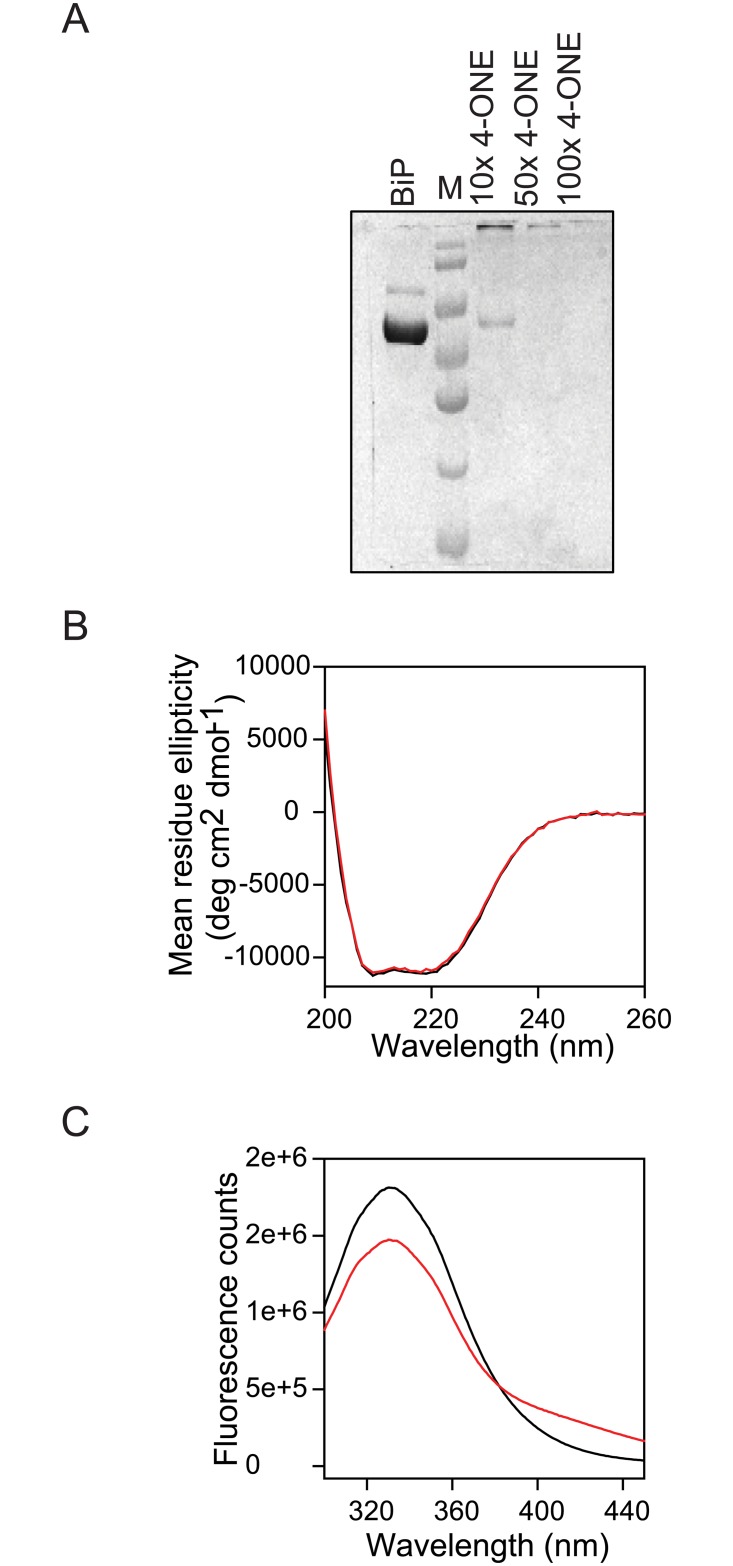
Structural characterization of the wild-type and chemically modified BiP proteins. (A) SDS-PAGE of wild-type and chemically modified BiP with increasing levels of 4-ONE. Increasing 4-ONE shows increasing loss of monomer protein. (B) Far-UV circular dichroism (CD) spectra of wild-type (black) and chemically modified BiP (red). (C) Fluorescence spectra of wild-type BiP (black) and chemically modified BiP (red).

BiP was subjected to chemical modification by 4-ONE by incubating overnight at room temperature, using the protocols described above. With 4-ONE, the adduct formation could be due to either through slow Michael’s addition to lysine, histidine and cysteine residues or the fast, much more common Schiff base addition to lysine residues [36,37]. The adducts were treated with 10 mM NaBH_4_ in order to stabilize both Michael and Schiff adducts with lysine by reductive trapping, which are otherwise known to be somewhat reversible [36,37]. Upon chemical modification, BiP did not form any visible aggregates. However, when modified BiP was loaded on the SDS-PAGE (Fig 3A), clear loss of monomer was observed with the formation of large, high molecular weight soluble aggregates (> 170 kDa) that were unable to enter the gel. Such monomer loss was observed at all the three concentration ratios (10X, 50X, and 100X) of 4-ONE to BiP. 4-ONE is known to be a strong crosslinking agent in proteins and two lysines are usually involved in this crosslinking reaction [37]. The crosslinking products are formed by Schiff base addition followed by ketoaldehyde-amine adduct formation [38].

### Chemical modification perturbed the tertiary structure of BiP, but not its secondary structure

Circular dichroism (CD) was used to probe the effect of chemical modification on the secondary structure of the protein. The CD spectra of unmodified BiP showed a combination of α- and β-structures (Fig 3B). The negative bands at 208 and 222 nm are characteristic of an α-helical structure [39]. The absence of a clear separation between the 208 and 222 nm bands indicates the presence of β-structure. For a β-structure, CD spectrum shows a -ve band at 216 nm. This far-UV CD spectrum of wild-type BiP matched with that published in the literature [40], and is also consistent with its crystal structure (Protein Data Bank ID: 5E84). More importantly, no change in the CD spectrum was observed upon chemical modification (Fig 3B), indicating that the modification did not significantly perturb the secondary structure of BiP.

Intrinsic fluorescence is often considered as a probe for the tertiary structure of proteins, which originates from the aromatic side chains of predominantly tryptophan residues [41]. BiP contains two tryptophans, Trp103 in the NBD and Trp600 in the SBD (Fig 1). Unmodified BiP showed a fluorescence spectrum with an emission maximum close to 331nm (Fig 2C), indicating that the two tryptophans are buried in the hydrophobic protein environment. In general, tryptophans exposed to aqueous environment exhibit a fluorescence maximum at 360 nm, whereas the tryptophans buried in the hydrophobic environment show an emission maximum close to 320 nm [41]. Upon chemical modification, a clear increase in fluorescence at red wavelengths was observed with the loss of fluorescence at blue wavelengths (with no change in the area under the curve). This fluorescence change indicates that at least one of the tryptophans is exposed to solvent upon chemical modification, implying partial unfolding of the protein. As discussed below in the section on mass spectrometry results, this tryptophan seems to be from the NBD with which chemically modified residues make critical contacts compared to the tryptophan in SBD.

### Chemical modification of BiP by 4-ONE resulted in the loss of protein stability

#### Chemical modification destabilizes the secondary structure of BiP

**Fig 4:**
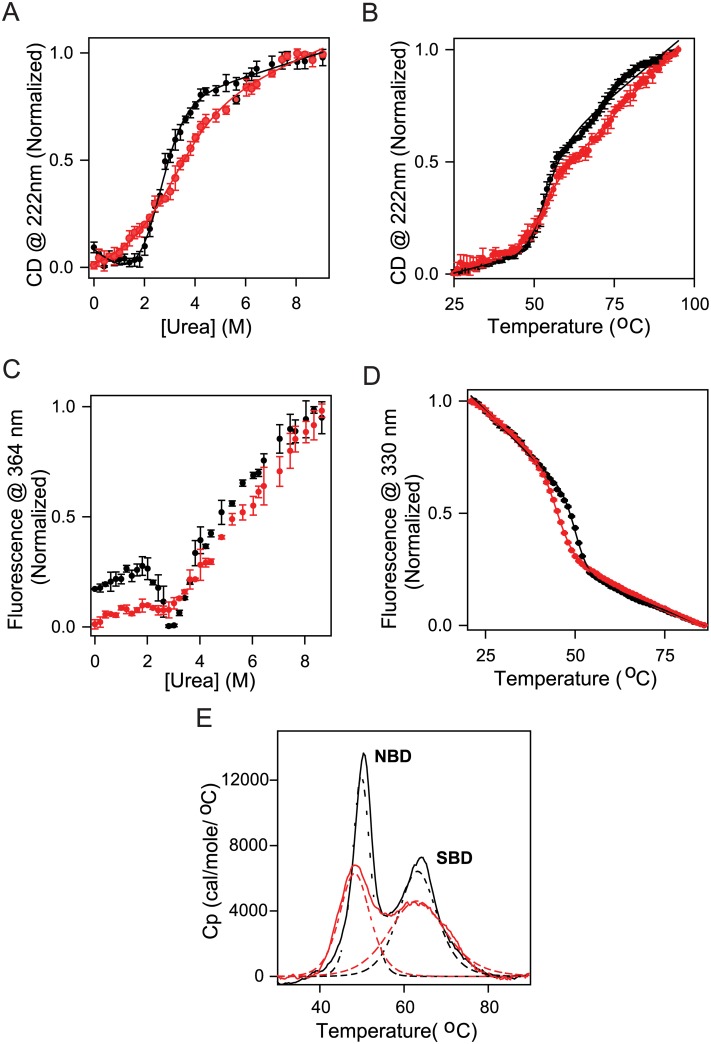
(A) Changes in the CD signal at 222 nm as a function of urea concentration. (B) Changes in the CD signal at 222 nm as a function of solution temperature. (C) Changes in the protein fluorescence at 364 nm as a function of urea concentration. (D) Changes in the protein fluorescence at 330 nm as a function of solution temperature. (E) Changes in the specific heat capacity of the protein as a function of increasing temperature as measured by DSC. The deconvolution fits are indicated as dashed lines. In all panels, black and red colors indicate the data for wild-type BiP and chemically modified BiP, respectively.

Equilibrium denaturant melts were used to probe the effect of chemical modification on the stability of BiP. CD signal was used to monitor the effect on the secondary structure of the protein. Urea was used as the denaturant to destabilize the protein structure. Both unmodified and modified BiP showed single, sigmoidal transitions (Fig 4A). However, the denaturant melt of modified BiP clearly deviates from that of the unmodified BiP, indicating that the chemical modification affected the stability of the secondary structure of BiP, although no changes in secondary structure were observed in the absence of denaturant upon chemical modification (Fig 3B). These denaturant melts were fit to a two-state equilibrium unfolding model to obtain the Gibbs free energy of unfolding, ΔG°_unf_, and the m-value, slope of the linear variation of ΔG_unf_ with denaturant concentration [42,43]. The fit parameters were ΔG°_unf_ = 2.88 ± 0.37 kcal/mol and m = -1.16 ± 0.11 (kcal/mol)/M [urea] for unmodified BiP, and ΔG°_unf_ = 0.51 ± 0.84 kcal/mol and m = -0.41 ± 0.13 (kcal/mol)/M [urea] for modified BiP. These values clearly indicate that the secondary structure of the protein is destabilized by ΔΔG°_unf_ = 2.37 ± 0.92 kcal/mol. Further, the decrease in the m-value indicates the increased population of partially unfolded states [44], implying that the chemical modification might be decreasing the stability of one or both the domains of BiP.

In the case of two-domain proteins such as BiP, the above fit ΔG°_unf_ values provide only qualitative information on whether the chemical modification is destabilizing the protein, but do not provide any quantitative information unless the two domains are unfolding as a single cooperative unit. The m-value obtained from denaturant melts is a measure of the difference in the accessible surface area (ASA) between the native and unfolded states [45]. From recently determined crystal structure of BiP [29], calculated ASA was 28671 Å^2^ (using the program Accelrys Discovery Studio Visualizer), which corresponds to a m-value of 3.52 (kcal/mol)/M [urea] (calculated using the empirical equation, *m*-value (cal/mol/M [urea]) = 374 + 0.11 (Δ*ASA*)) [45]. This calculated m-value is clearly higher than the measured m-value for wild-type BiP (Fig 4A), indicating that the two domains NBD and SBD do not unfold as a single cooperative unit. Therefore, the obtained ΔG°_unf_ values from fitting denaturant melts should only be used to probe the qualitative effect of chemical modification on BiP stability.

In addition to denaturant melts, thermal melts were used to measure the effect of chemical modification on protein stability (Fig 4B). In thermal melts, increase in temperature was used as the denaturant. With increase in temperature, modified BiP unfolded at lower temperatures (with a midpoint transition temperature, T_m_ = 51.6 ± 0.5°C) compared to the wild-type BiP (T_m_ = 53.1 ± 0.4°C). Although this change in T_m_ seems to be marginal, it is quite reproducible as evident from the errors on the measured T_m_ values. Such minor changes in T_m_ values have been seen earlier in other proteins, and have been shown to significantly affect protein function [46,47]. Similar to denaturant melts, a decrease in the slope of CD signal with temperature was observed in the case of modified BiP, indicating that the chemical modification might be destabilizing one or both domains of BiP.

#### Chemical modification destabilizes the tertiary structure of BiP

We used denaturant and thermal melts with fluorescence as the signal to measure the effect of chemical modification on the tertiary structure of BiP. In denaturant melts, a clear difference was observed at low denaturant concentrations between the wild-type and chemically modified BiP. Wild-type protein populates a partially unfolded state at 2.7 M urea concentration that has the same fluorescence properties as that of the chemically modified BiP in the absence of denaturant (Fig 4C). This is quite consistent with the effect of chemical modification on the fluorescence spectrum (Fig 3C), which indicates an increased population of a partially unfolded state upon chemical modification. The latter part of the denaturant melts (> 2.7 M [urea]) was very similar for the wild-type and chemically modified BiP.

When the same tertiary structure was monitored using thermal melts, chemically modified BiP unfolds at lower temperatures compared to wild-type BiP. The midpoint melting temperatures (T_m_) for wild-type and chemical modified BiP proteins were 50.5 ± 0.1°C and 46.2 ± 0.2°C, respectively, indicating that the chemical modification destabilized one or both domains of BiP.

#### Chemical modification destabilized predominantly the NBD compared to the SBD

For a multi-domain protein such as BiP, differential scanning calorimetry (DSC) provides a much better information about the unfolding of individual domains because of their intrinsic differences in the heat capacities [48]. BiP shows two clear, well-resolved peaks that correspond to the two domains in the protein (Fig 4E). These two domains have been identified earlier as that of NBD and SBD [49], with NBD unfolding with a T_m_ of 50.3 ± 0.1°C whereas the SBD unfolding with a Tm of 63.7 ± 0.1°C. Upon chemical modification, a significant decrease in the T_m_ value of NBD was observed (T_m_ = 48.3 ± 0.1°C), compared to relatively no change in the T_m_ value of SBD (T_m_ = 63.3 ± 0.2°C) (Fig 4E). These results indicate that the chemical modification predominantly destabilizes the NBD compared to SBD.

The above observation from DSC measurements that chemical modification predominantly destabilizes the NBD compared to SBD is also consistent with the denaturant and thermal melts discussed above. The effect of chemical modification is clearly on the NBD which unfolds at lower denaturant concentration (Fig 4C). In thermal melts (Fig 4D), chemical modification affects only the transition that is occurring below 55°C, which corresponds to the NBD, with no effect on the signal at higher temperatures indicating that the chemical modification does not significantly affect the SBD.

### Chemical modification of BiP affected its ATP binding and ATP hydrolysis, but not its immunoglobulin binding and non-specific chaperone activity

BiP has several functional roles and is known to be the master regulator of ER homeostasis [50]. Some key roles include (1) ATP dependent active folding of its substrates, and (2) ATP independent non-specific chaperone activity of rescuing proteins from aggregation. In ATP-dependent BiP function, ATP binding, its hydrolysis, and substrate binding are three key steps [31]. We examined the effect of chemical modification on these processes.

#### Modified BiP binds to ATP less efficiently compared to the unmodified BiP

**Fig 5:**
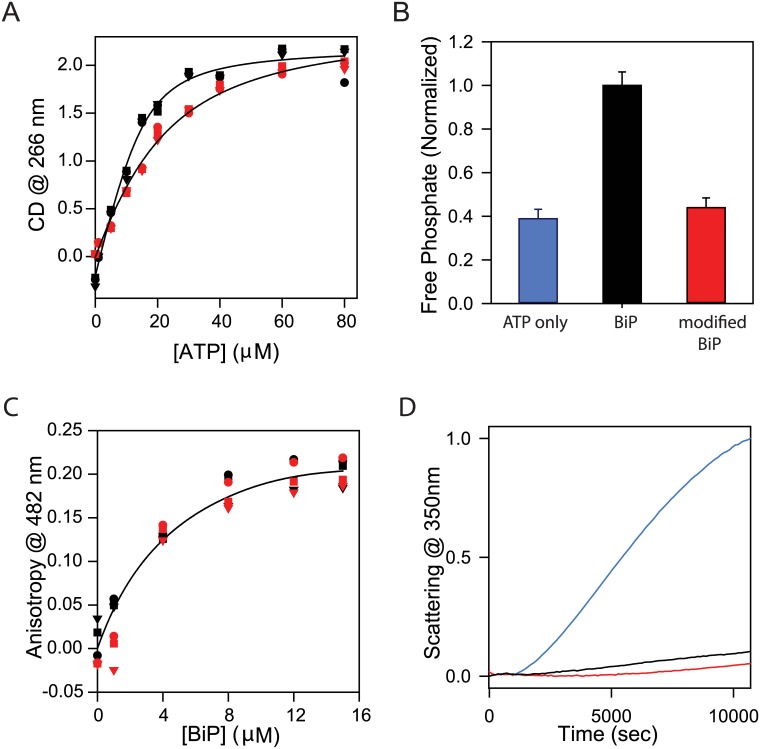
(A) Changes in the near-UV CD signal of BiP at 266nm as a function of varying ATP concentration. (B) Changes in ATPase activity of protein before and after chemical modification. Blue bar is ATP only. (C) Changes in fluorescence anisotropy of peptide HTFPAVL at 482nm as a function of varying BiP concentration. (D) Changes in light scattering at 350 nm due to the aggregation of citrate synthase as a function of incubation time at 43°C. Blue indicates citrate synthase alone. In all panels, black and red colors indicate the data for wild-type BiP and chemically modified BiP, respectively.

Changes in the near-UV CD signal of the BiP protein at 266 nm were used to monitor the effect of chemical modification on its ATP binding affinity [49]. At this wavelength, the major contribution to the signal is due to the aromatic side chains of phenylalanine residues. Maximal difference in the CD signal was seen at 266nm before and after binding to ATP. With increasing concentrations of ATP, the near-UV CD signal of BiP changes because of the tertiary structural change in the nucleotide binding pocket of BiP, reaching a plateau indicating a saturation of the NBD by ATP (Fig 5A). This binding data were fitted to obtain the K_d_ values using Eqs 3 and 6 described in Methods section. Chemically modified BiP binds to ATP with a K_d_ value that is 4.4 times the K_d_ value of the unmodified BiP. The K_d_ values were 2.9 μM ± 0.5 μM for unmodified BiP and 12.9 μM ± 1.5 μM for chemically modified BiP. Since binding affinity is the inverse of K_d_, these K_d_ values indicate that the BiP significantly loses its ability to bind to ATP when chemically modified with 4- ONE. This change in the K_d_ values also correlates with the loss of stability of NBD (Fig 4). Upon chemical modification, NBD becomes less stable and binds to ATP less effectively compared to the unmodified BiP.

#### Modified BiP loses its ATPase activity compared to the unmodified BiP

Malachite green dye binding assay was used to measure the ATPase activity of BiP [51,52]. The free phosphate generated from ATP hydrolysis forms a complex with malachite green and ammonium molybdate, and can be quantified by measuring its absorbance at 660 nm. The ability of wild-type and chemically modified BiP to generate free phosphate from ATP was monitored over a period of time. After 50 minutes, ATPase activity of the chemically modified BiP was 60% lower than that of the unmodified BiP (Fig 5B), and was close to that of the free ATP hydrolysis in the absence of BiP. This data shows that the chemical modification of BiP by 4-ONE results in the complete loss of its ATPase activity.

#### Modified BiP binds to its substrate immunoglobulin peptide as efficiently as that of the unmodified BiP

Immunoglobulins are the specific substrates for BiP [33]. Earlier studies have identified the specific protein regions in immunoglobulins that are involved in binding to BiP [53–55]. We used one such peptide HTFPAVLGSC derived from the CH1 domains of immunoglobulin to measure the effect of chemical modification. This peptide has been used previously by other research groups to measure the effect of BiP mutations on immunoglobulin binding [31]. We labelled the peptide with a fluorescent dye lucifer yellow iodoacetamide. The dye is fluorescent only when it is covalently attached to a thiol group. Its fluorescence is quenched by the iodoacetamide group in its free form. We examined the changes in the anisotropy of the peptide upon binding to BiP (Fig 5C). In the unbound form, the peptide is free to tumble leading to near-zero anisotropy. Upon binding, the peptide has restricted rotational freedom in the binding pocket, and hence results in an increased anisotropy (Fig 5C). The changes in the fluorescence anisotropy as a function of the concentration of BiP were identical for wild-type and modified proteins, indicating that the chemical modification of BiP by 4-ONE does not affect its binding to immunoglobulins.

#### Modified BiP has similar chaperone activity as that of unmodified BiP

Citrate synthase (CS) is a non-specific substrate protein that is commonly used to test the aggregation suppressor activity of chaperones such as BiP, GroEL, and others [56–58]. This assay of rescuing of non-specific proteins such as citrate synthase from heat-induced aggregation by chaperones does not require ATP [59,60]. Under isothermal conditions at 43°C, CS aggregates, and a chaperone that is an aggregation suppressor prevents such aggregation of CS. This is clearly evident from the aggregation kinetics of CS in the absence and presence of wild-type BiP (Fig 5D). Aggregation was monitored by changes in light scattering due to increased solution turbidity. In the presence of BiP, CS aggregates minimally compared to that in the absence of BiP. Similar aggregation suppressor activity has also been seen for chemically modified BiP (Fig 5D). This shows that the chemical modification of BiP by 4-ONE did not significantly affect the aggregation suppressor activity of BiP.

### 4-ONE modifies key lysine residues in BiP involved in ATP binding and hydrolysis

**Fig 6:**
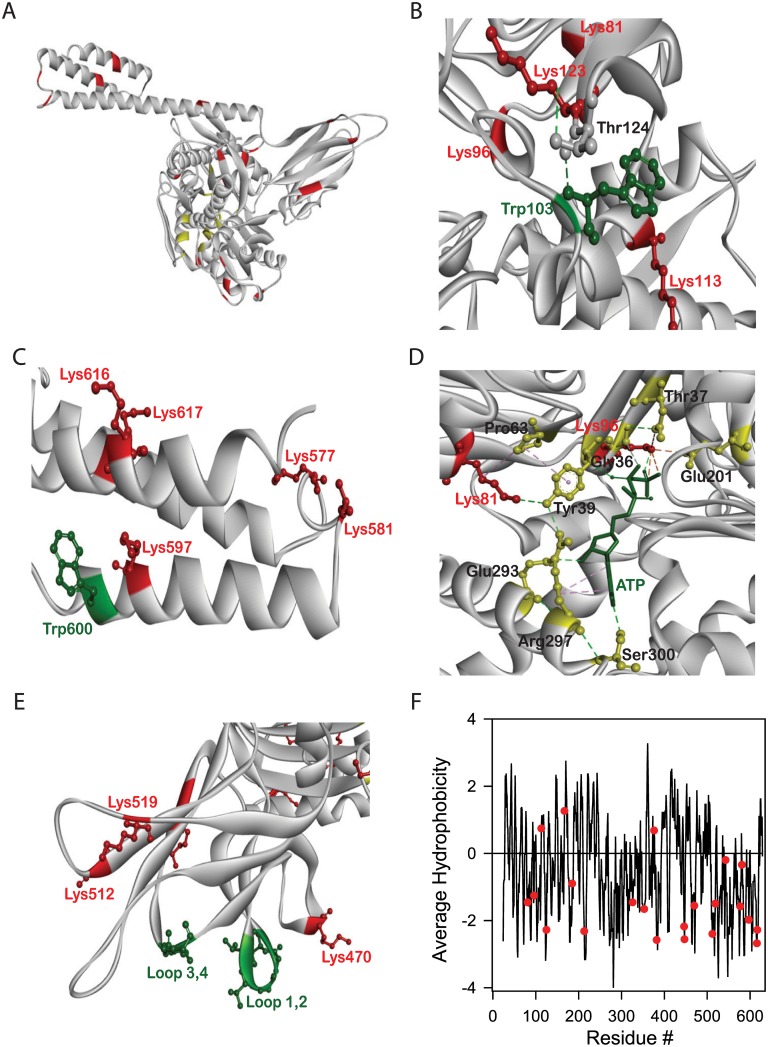
(A) Crystal structure of BiP indicating all chemically modified residues (red) and residues in ATP binding pocket (yellow). (B) Interactions between Trp103 and chemically modified Lys123 in NBD. (C) Trp600 in SBD does not form any interactions with chemically modified lysines. (D) Nucleotide binding domain (NBD) showing stabilizing interactions between key residues in the ATP binding pocket (yellow) and chemically modified lysine residues (red). (E) Substrate binding domain (SBD-β) showing no interactions between the loops responsible for immunoglobulin binding (green) and chemically modified residues (red). (F) 5-residue average hydrophobicity plot (calculated using the program Accelrys Discovery Studio Visualizer) of BiP indicating the location of chemically modified residues (red).

Above results of the effect of chemical modification on the structure, stability and function of BiP indicate that the major effect is on the nucleotide binding domain (NBD) where ATP binds rather than the substrate binding domain (SBD) where immunoglobulin binds. To understand the underlying mechanism, we examined the specific amino acid residues that were modified by 4-ONE. Using LC-MS analysis on trypsin digested protein samples and peptide mapping, we found 21 lysine residues and 1 histidine residue that were significantly modified by 4-ONE (Fig 6A). Many of these modifications were found in the NBD. This is again consistent with the decreased stability of NBD compared to SBD after chemical modification (Fig 4E). The domain which is less stable is more prone to increased partial unfolding leading to an increased exposure of protein regions to the solvent, and hence is more susceptible to chemical modifications [61]. In addition, many chemically modified lysine residues were close to the single tryptophan residue (Trp103) in NBD. Trp103 forms a stabilizing hydrogen bond network with Lys123 and Thr124 (Fig 6B). Lys123 is one of the highly chemically modified lysines, indicating that it is quite likely that the chemical modification destabilizes the structure around Trp103 leading to its partial exposure to solvent. No such stabilizing interactions were seen between the tryptophan residue (Trp600) in SBD and modified Lys residues (Fig 6C). This explains why the chemical modification leads to an increase in fluorescence at red wavelengths (Fig 3C), and why chemical modification specifically destabilizes the tertiary structure around the tryptophan residue in the NBD (Fig 4C & 4D).

In terms of the effect of chemical modification on the ATP binding and hydrolysis, two key lysine residues that were chemically modified by 4-ONE, Lys81 and Lys96, were part of the stabilizing network of amino acid interactions involved in ATP binding and hydrolysis. Recently determined crystal structure of BiP (Protein Data Bank ID: 5E84) indicates that Lys81 forms direct interactions with Tyr39 and Pro63, whereas Lys96 interacts directly with Glu201 and the two oxygens of the gamma phosphate of ATP (Fig 6D). Amide side chain of Lys81 forms a hydrogen bond with the side chain hydroxyl group of Tyr39 (with oxygen as the acceptor). In addition, the four methylene groups of Lys81 are involved in hydrophobic interactions with Pro63. Similar hydrophobic interactions between lysines and prolines have been observed earlier in other proteins [62]. Pro63 hydrophobically interacts with Tyr39. Main chain amide of Tyr39 forms a hydrogen bond with an oxygen attached to the beta-phosphate group of ATP. The side chain hydroxyl group of Tyr39 is hydrogen bonded with the amide side chain of Arg297. Arg297 stabilizes ATP binding through a pi-alkyl interaction with the adenine ring of ATP. Amide side chain of Arg297 forms a hydrogen bond with the carbonyl oxygen of Glu293, which is further hydrogen bonded with the hydroxyl group of ribose ring of ATP. In addition, the carbonyl oxygen of Arg297 participates in a hydrogen bond with the main chain amide of Ser300, which stabilizes ATP binding by interacting with its adenine ring. With respect to the interactions made by Lys96, its side chain forms hydrogen bonds with the two oxygens of the terminal gamma-phosphate group of ATP (Fig 6D). Further, Lys96 interacts with Glu201 through a salt bridge, which is critical for the ATPase activity of BiP [63,64]. In addition, Lys96 side chain participates in an electrostatic interaction with the carbonyl oxygen of Asp34. Asp34 side chain forms a hydrogen bond with the main chain amide of Gly36, which interacts with ATP via a weak carbon-hydrogen bond. Further, earlier studies have shown that Lys71 in bovine HSP70 that occurs at an analogous position to Lys96 in BiP is essential for ATP hydrolysis [65]. Because of these various stabilizing interactions formed by Lys81 and Lys96 in the ATP binding pocket, chemical modification of these two key lysine residues appears to break the stabilizing interaction network of amino acids involved in ATP binding and hydrolysis, thus leading to a drastic decrease in ATP binding and complete loss of ATP hydrolysis.

In terms of immunoglobulin binding, none of the lysine residues that were modified by 4-ONE are part of the hydrophobic network of amino acids that interact with immunoglobulin in SBD (Fig 6E). BiP binds to polypeptides using hydrophobic interactions, in particular, hydrophobic amino acids in the two loops labeled L_12_ and L_34_ [29]. None of the chemically modified lysine residues interact with these two loops (Fig 6E). This might be the reason why chemical modification did not affect the BiP binding to immunoglobulin peptide.

In terms of the effect of chemical modification on the non-specific aggregation suppressor role of BiP, which occurs through nonspecific hydrophobic interactions between the misfolded proteins and BiP, only 3 out of 21 chemically modified lysines occur predominantly in the hydrophobic regions of the protein (Fig 6F). Such minor modifications in hydrophobic amino acid patches in BiP are not expected to significantly affect its non-specific aggregation suppressor activity.

## Discussion

Free radical damage is a major cause of the loss of cellular functions, and occurs in response to various factors, such as stress, ageing, pollution, smoking, and alcohol [66]. In addition, increased presence of free radicals has been shown to trigger various diseases [21–23]. Free radicals attack lipid cell membranes, and generate reactive species such as 4-oxononenal (4-ONE) [24–26]. These chemical agents modify various key proteins involved in cellular function [28,67–72]. Using an alcohol diver disease (ALD) mouse model, our colleagues, Dennis Petersen and his lab, have identified 8 key proteins that were modified by lipid peroxidation products [28]. BiP is one such key protein. Because of the importance of BiP in cellular homeostasis, unfolded protein response, and in rescuing misfolded proteins from aggregation, it is important to understand how chemical modification of BiP affects its structure and function.

Earlier study by Dennis Petersen’s lab probed the effects of chemical modification on some of the functional properties of BiP [67], in particular on the ATP hydrolysis and non-specific aggregation suppressor role of BiP, and proposed the underlying hypotheses based on a computational modeling of the protein structure. In this manuscript, we examined the effect of chemical modifications on the structure, stability, and some of the functional properties that were not previously examined in the earlier publication [67], in particular, on the ATP binding and on the binding of immunoglobulin, which is the specific substrate for BiP, and more importantly the changes in protein structure and stability upon chemical modification. The results presented here provided a clear picture of how changes in protein function can be explained in terms of the changes in protein structure and stability. In addition, recently determined crystal structure of BiP [29] after the initial publication [67] provided an added advantage in terms of linking the location of chemical modifications in protein structure to changes in protein stability and function.

Chemical modification of BiP by 4-ONE resulted in the formation of soluble, irreversible aggregates because of specific crosslinking of lysine residues of different monomers (Figs 3A and 6A). The chemical modification did not perturb the α/β secondary structure of BiP with no change in the far-UV CD spectrum (Fig 3B). However, the modification affected the tertiary structure in terms of increase in fluorescence at red wavelengths (Fig 3C). Identifying the chemically modified amino acids in the molecular structure of BiP (Fig 6A) and interpreting chemical and thermal denaturation experiments (Fig 4) based on the location of modified residues in protein structure indicate that the domain that is predominantly destabilized is the NBD rather than the SBD. This is also consistent with the DSC results where significant destabilization has been observed in the case of NBD rather than the SBD (Fig 4E). In terms of function, chemical modification decreased the ATP binding affinity (Fig 5A) and resulted in the complete loss of ATP hydrolysis (Fig 5B). This loss in function is in correlation with the loss of stability of NBD (Fig 4). In addition, some of the chemically modified lysine residues make critical contacts with the ATP and the amino acids involved in ATP binding and ATP hydrolysis (Fig 6D). Contrary to ATP binding and hydrolysis, chemical modification did not affect BiP binding to immunoglobulin CH1 fragment (Fig 5C), because the modified lysine residues are not part of the two hydrophobic loops in SBD that are involved in immunoglobulin binding (Fig 6E). Also, chemical modification did not affect BiP rescuing non-specific proteins such as citrate synthase from aggregation (Fig 5D), because such function is mediated by hydrophobic interactions between BiP and substrate proteins, and lysines are not hydrophobic amino acids. Taken together, all the above results on the effect of chemical modifications on the function of BiP show a direct correlation in terms of the changes in the structure and stability of BiP. Such a clear correlation between the protein structure, stability and function upon chemical modification has not been shown before.

## References

[1] Fink AL. Chaperone-mediated protein folding. 1999; Physiol Rev 79: 425–449. 10221986 10.1152/physrev.1999.79.2.425

[2] Bukau B, Horwich AL. The Hsp70 and Hsp60 chaperone machines. 1998; Cell 92: 351–366. 9476895 10.1016/s0092-8674(00)80928-9

[3] Zhang K, Kaufman RJ. The unfolded protein response: a stress signaling pathway critical for health and disease. 2006; Neurology 66: S102–109. doi: 10.1212/01.wnl.0000192306.98198.ec 16432136 10.1212/01.wnl.0000192306.98198.ec

[4] Naidoo N. ER and aging-Protein folding and the ER stress response. 2009; Ageing Res Rev 8: 150–159. 19491040 10.1016/j.arr.2009.03.001

[5] Toledo H, Carlino A, Vidal V, Redfield B, Nettleton MY, Kochan JP, et al Dissociation of glucose-regulated protein Grp78 and Grp78-IgE Fc complexes by ATP. 1993; Proc Natl Acad Sci U S A 90: 2505–2508. 8460165 10.1073/pnas.90.6.2505 PMC46116

[6] Stevens FJ, Argon Y. Protein folding in the ER. 1999; Semin Cell Dev Biol 10: 443–454. doi: 10.1006/scdb.1999.0315 10597627 10.1006/scdb.1999.0315

[7] Bukau B, Weissman J, Horwich A. Molecular chaperones and protein quality control. 2006; Cell 125: 443–451. doi: 10.1016/j.cell.2006.04.014 16678092 10.1016/j.cell.2006.04.014

[8] Hendershot L, Bole D, Kearney JF. The role of immunoglobulin heavy chain binding protein in immunoglobulin transport. 1987; Immunol Today 8: 111–114. doi: 10.1016/0167-5699(87)90861-9 25289812 10.1016/0167-5699(87)90861-9

[9] Lee YK, Brewer JW, Hellman R, Hendershot LM. BiP and immunoglobulin light chain cooperate to control the folding of heavy chain and ensure the fidelity of immunoglobulin assembly. 1999; Mol Biol Cell 10: 2209–2219. 10397760 10.1091/mbc.10.7.2209 PMC25436

[10] Knittler MR, Haas IG. Interaction of BiP with newly synthesized immunoglobulin light chain molecules: cycles of sequential binding and release. 1992; EMBO J 11: 1573–1581. 1563355 10.1002/j.1460-2075.1992.tb05202.x PMC556606

[11] Guerriero CJ, Brodsky JL. The delicate balance between secreted protein folding and endoplasmic reticulum-associated degradation in human physiology. 2012; Physiol Rev 92: 537–576. doi: 10.1152/physrev.00027.2011 22535891 10.1152/physrev.00027.2011 PMC4162396

[12] Luo S, Mao C, Lee B, Lee AS. GRP78/BiP is required for cell proliferation and protecting the inner cell mass from apoptosis during early mouse embryonic development. 2006; Mol Cell Biol 26: 5688–5697. doi: 10.1128/MCB.00779-06 16847323 10.1128/MCB.00779-06 PMC1592753

[13] Katanasaka Y, Ishii T, Asai T, Naitou H, Maeda N, Koizumi F, et al Cancer antineovascular therapy with liposome drug delivery systems targeted to BiP/GRP78. 2010; Int J Cancer 127: 2685–2698. doi: 10.1002/ijc.25276 20178102 10.1002/ijc.25276

[14] Li Z, Li Z. Glucose regulated protein 78: a critical link between tumor microenvironment and cancer hallmarks. 2012; Biochim Biophys Acta 1826: 13–22. doi: 10.1016/j.bbcan.2012.02.001 22426159 10.1016/j.bbcan.2012.02.001

[15] Gorbatyuk MS, Gorbatyuk OS. The molecular chaperone GRP78/BiP as a therapeutic target for neurodegenerative disorders: A mini review. 2013; J Genet Syndr Gene Ther 4. 10.4172/2157-7412.1000128 PMC3674964 23750325

[16] Wang M, Wey S, Zhang Y, Ye R, Lee AS. Role of the unfolded protein response regulator GRP78/BiP in development, cancer, and neurological disorders. 2009; Antioxid Redox Signal 11: 2307–2316. doi: 10.1089/ARS.2009.2485 19309259 10.1089/ars.2009.2485 PMC2819800

[17] Lee AS. Glucose-regulated proteins in cancer: molecular mechanisms and therapeutic potential. 2014; Nat Rev Cancer 14: 263–276. doi: 10.1038/nrc3701 24658275 10.1038/nrc3701 PMC4158750

[18] Pfaffenbach KT, Lee AS. The critical role of GRP78 in physiologic and pathologic stress. 2011; Curr Opin Cell Biol 23: 150–156. doi: 10.1016/j.ceb.2010.09.007 20970977 10.1016/j.ceb.2010.09.007 PMC3043145

[19] Ni M, Zhang Y, Lee AS. Beyond the endoplasmic reticulum: atypical GRP78 in cell viability, signalling and therapeutic targeting. 2011; Biochem J 434: 181–188. doi: 10.1042/BJ20101569 21309747 10.1042/BJ20101569 PMC3353658

[20] Lee AS. The glucose-regulated proteins: stress induction and clinical applications. 2001; Trends Biochem Sci 26: 504–510. 11504627 10.1016/s0968-0004(01)01908-9

[21] Halliwell B. Lipid peroxidation in vivo and in vitro in relation to atherosclerosis: some fundamental questions. 1988; Agents Actions Suppl 26: 223–231. 3064570

[22] Halliwell B, Chirico S. Lipid peroxidation: its mechanism, measurement, and significance. 1993; Am J Clin Nutr 57: 715S–724S; discussion 724S-725S. 8475889 10.1093/ajcn/57.5.715S

[23] Uchida K. 4-Hydroxy-2-nonenal: a product and mediator of oxidative stress. 2003; Prog Lipid Res 42: 318–343. 12689622 10.1016/s0163-7827(03)00014-6

[24] Fritz KS, Petersen DR. Exploring the biology of lipid peroxidation-derived protein carbonylation. 2011; Chem Res Toxicol 24: 1411–1419. doi: 10.1021/tx200169n 21812433 10.1021/tx200169n PMC3178011

[25] Petersen DR, Doorn JA. Reactions of 4-hydroxynonenal with proteins and cellular targets. 2004; Free Radic Biol Med 37: 937–945. doi: 10.1016/j.freeradbiomed.2004.06.012 15336309 10.1016/j.freeradbiomed.2004.06.012

[26] Esterbauer H, Schaur RJ, Zollner H. Chemistry and biochemistry of 4-hydroxynonenal, malonaldehyde and related aldehydes. 1991; Free Radic Biol Med 11: 81–128. 1937131 10.1016/0891-5849(91)90192-6

[27] Fritz KS, Petersen DR. An overview of the chemistry and biology of reactive aldehydes. 2013; Free Radic Biol Med 59: 85–91. doi: 10.1016/j.freeradbiomed.2012.06.025 22750507 10.1016/j.freeradbiomed.2012.06.025 PMC3540155

[28] Smathers RL, Galligan JJ, Stewart BJ, Petersen DR. Overview of lipid peroxidation products and hepatic protein modification in alcoholic liver disease. 2011; Chem Biol Interact 192: 107–112. doi: 10.1016/j.cbi.2011.02.021 21354120 10.1016/j.cbi.2011.02.021 PMC3109208

[29] Yang J, Nune M, Zong Y, Zhou L, Liu Q. Close and allosteric opening of the polypeptide-binding site in a human Hsp70 chaperone BiP. 2015; Structure 23: 2191–2203. doi: 10.1016/j.str.2015.10.012 26655470 10.1016/j.str.2015.10.012 PMC4680848

[30] Arakawa A, Handa N, Shirouzu M, Yokoyama S. Biochemical and structural studies on the high affinity of Hsp70 for ADP. 2011; Protein Sci 20: 1367–1379. doi: 10.1002/pro.663 21608060 10.1002/pro.663 PMC3189522

[31] Marcinowski M, Holler M, Feige MJ, Baerend D, Lamb DC, Buchner J. Substrate discrimination of the chaperone BiP by autonomous and cochaperone-regulated conformational transitions. 2011; Nat Struct Mol Biol 18: 150–158. doi: 10.1038/nsmb.1970 21217698 10.1038/nsmb.1970

[32] Young JC. Mechanisms of the Hsp70 chaperone system. 2010; Biochem Cell Biol 88: 291–300. doi: 10.1139/o09-175 20453930 10.1139/o09-175 PMC5026485

[33] Mayer M, Reinstein J, Buchner J. Modulation of the ATPase cycle of BiP by peptides and proteins. 2003; J Mol Biol 330: 137–144. 12818208 10.1016/s0022-2836(03)00556-4

[34] Pace CN. Determination and analysis of urea and guanidine hydrochloride denaturation curves. 1986; Methods Enzymol 131: 266–280. 3773761 10.1016/0076-6879(86)31045-0

[35] Tran TM, Satumtira N, Dorris ML, May E, Wang A, Furuta E, et al HLA-B27 in transgenic rats forms disulfide-linked heavy chain oligomers and multimers that bind to the chaperone BiP. 2004; J Immunol 172: 5110–5119. 15067095 10.4049/jimmunol.172.8.5110

[36] Lin D, Sayre LM (2008) Lipoxidation-Derived Electrophiles as Biological Reactive Intermediates In: Elfarra A, editor. Advances in Bioactivation Research. New York, NY: Springer New York pp. 1–34.

[37] Cai J, Hill BG, Bhatnagar A, Pierce WM, Prough RA (2008) Bioactivation and protein modification reactions of unsaturated aldehydes In: Elfarra A, editor. Advances in Bioactivation Research. New York, NY: Springer New York pp. 1–21.

[38] Xu G, Sayre LM. Structural elucidation of a 2:2 4-ketoaldehyde−amine adduct as a model for lysine-directed cross-linking of proteins by 4-ketoaldehydes. 1999; Chemical Research in Toxicology 12: 862–868. doi: 10.1021/tx9900573 10490509 10.1021/tx9900573

[39] Greenfield NJ. Using circular dichroism spectra to estimate protein secondary structure. 2006; Nature Protoc 6: 2876–2890. 10.1038/nprot.2006.202 PMC2728378 17406547

[40] Rosenes Z, Mulhern TD, Hatters DM, Ilag LL, Power BE, Hosking C, et al The anti-cancer IgM monoclonal antibody PAT-SM6 binds with high avidity to the unfolded protein response regulator GRP78. 2012; PLoS One 7: e44927 doi: 10.1371/journal.pone.0044927 23028685 10.1371/journal.pone.0044927 PMC3446985

[41] Lakowicz JR (2006) Principles of Fluorescence Spectroscopy. New York: Springer Science.

[42] Santoro MM, Bolen DW. A test of the linear extrapolation of unfolding free energy changes over an extended denaturant concentration range. 1992; Biochemistry 31: 4901–4907. 1591250 10.1021/bi00135a022

[43] Santoro MM, Bolen DW. Unfolding free energy changes determined by the linear extrapolation method. 1. Unfolding of phenylmethanesulfonyl alpha-chymotrypsin using different denaturants. 1988; Biochemistry 27: 8063–8068. 3233195 10.1021/bi00421a014

[44] Soulages JL. Chemical denaturation: Potential impact of undetected intermediates in the free energy of unfolding and m-values obtained from a two-state assumption. 1998; Biophys J 75: 484–492. doi: 10.1016/S0006-3495(98)77537-X 9649410 10.1016/S0006-3495(98)77537-X PMC1299722

[45] Myers JK, Pace CN, Scholtz JM. Denaturant m values and heat capacity changes: relation to changes in accessible surface areas of protein unfolding. 1995; Protein Sci 4: 2138–2148. doi: 10.1002/pro.5560041020 8535251 10.1002/pro.5560041020 PMC2142997

[46] Arthur KK, Dinh N, Gabrielson JP. Technical decision making with higher order structure data: utilization of differential scanning calorimetry to elucidate critical protein structural changes resulting from oxidation. 2015; J Pharm Sci 104: 1548–1554. doi: 10.1002/jps.24313 25561411 10.1002/jps.24313

[47] Gersting SW, Kemter KF, Staudigl M, Messing DD, Danecka MK, Lagler FB, et al Loss of function in phenylketonuria is caused by impaired molecular motions and conformational instability. 2008; Am J Hum Genet 83: 5–17. doi: 10.1016/j.ajhg.2008.05.013 18538294 10.1016/j.ajhg.2008.05.013 PMC2443833

[48] Wen J, Arthur K, Chemmalil L, Muzammil S, Gabrielson J, Jiang Y. Applications of differential scanning calorimetry for thermal stability analysis of proteins: qualification of DSC. 2012; J Pharm Sci 101: 955–964. doi: 10.1002/jps.22820 22147423 10.1002/jps.22820

[49] Lamb HK, Mee C, Xu W, Liu L, Blond S, Cooper A, et al The affinity of a major Ca2+ binding site on GRP78 is differentially enhanced by ADP and ATP. 2006; J Biol Chem 281: 8796–8805. doi: 10.1074/jbc.M503964200 16418174 10.1074/jbc.M503964200

[50] Park KW, Eun Kim G, Morales R, Moda F, Moreno-Gonzalez I, Concha-Marambio L, et al The endoplasmic reticulum chaperone GRP78/BiP modulates prion propagation in vitro and in vivo. 2017; Sci Rep 7: 44723 doi: 10.1038/srep44723 28333162 10.1038/srep44723 PMC5363067

[51] Feng J, Chen Y, Pu J, Yang X, Zhang C, Zhu S, et al An improved malachite green assay of phosphate: mechanism and application. 2011; Anal Biochem 409: 144–149. doi: 10.1016/j.ab.2010.10.025 20971056 10.1016/j.ab.2010.10.025

[52] Geladopoulos TP, Sotiroudis TG, Evangelopoulos AE. A malachite green colorimetric assay for protein phosphatase activity. 1991; Anal Biochem 192: 112–116. 1646572 10.1016/0003-2697(91)90194-x

[53] Feige MJ, Hendershot LM, Buchner J. How antibodies fold. 2010; Trends Biochem Sci 35: 189–198. doi: 10.1016/j.tibs.2009.11.005 20022755 10.1016/j.tibs.2009.11.005 PMC4716677

[54] Gething MJ, Blond-Elguindi S, Buchner J, Fourie A, Knarr G, Modrow S, et al Binding sites for Hsp70 molecular chaperones in natural proteins. 1995; Cold Spring Harb Symp Quant Biol 60: 417–428. 8824415 10.1101/sqb.1995.060.01.046

[55] Knarr G, Gething MJ, Modrow S, Buchner J. BiP binding sequences in antibodies. 1995; J Biol Chem 270: 27589–27594. 7499221 10.1074/jbc.270.46.27589

[56] Buchner J, Schmidt M, Fuchs M, Jaenicke R, Rudolph R, Schmid FX, et al GroE facilitates refolding of citrate synthase by suppressing aggregation. 1991; Biochemistry 30: 1586–1591. 1671555 10.1021/bi00220a020

[57] Daugherty DL, Rozema D, Hanson PE, Gellman SH. Artificial chaperone-assisted refolding of citrate synthase. 1998; J Biol Chem 273: 33961–33971. 9852049 10.1074/jbc.273.51.33961

[58] Shao F, Bader MW, Jakob U, Bardwell JC. DsbG, a protein disulfide isomerase with chaperone activity. 2000; J Biol Chem 275: 13349–13352. 10788443 10.1074/jbc.275.18.13349

[59] Buchner J, Grallert H, Jakob U. Analysis of chaperone function using citrate synthase as nonnative substrate protein. 1998; Methods in Enzymology 290: 323–338. 9534173 10.1016/s0076-6879(98)90029-5

[60] Stronge VS, Saito Y, Ihara Y, Williams DB. Relationship between calnexin and BiP in suppressing aggregation and promoting refolding of protein and glycoprotein substrates. 2001; J Biol Chem 276: 39779–39787. doi: 10.1074/jbc.M107091200 11514579 10.1074/jbc.M107091200

[61] Manning MC, Chou DK, Murphy BM, Payne RW, Katayama DS. Stability of protein pharmaceuticals: an update. 2010; Pharm Res 27: 544–575. doi: 10.1007/s11095-009-0045-6 20143256 10.1007/s11095-009-0045-6

[62] Tanford C. Contribution of hydrophobic interactions to the stability of the globular conformation of proteins. 1962; J Am Chem Soc 84: 4240–4247.

[63] Gaut JR, Hendershot LM. Mutations within the nucleotide binding site of immunoglobulin-binding protein inhibit ATPase activity and interfere with release of immunoglobulin heavy chain. 1993; J Biol Chem 268: 7248–7255. 8463260

[64] Wei J, Gaut JR, Hendershot LM. In vitro dissociation of BiP-peptide complexes requires a conformational change in BiP after ATP binding but does not require ATP hydrolysis. 1995; J Biol Chem 270: 26677–26682. 7592894 10.1074/jbc.270.44.26677

[65] O'Brien MC, Flaherty KM, McKay DB. Lysine 71 of the chaperone protein Hsc70 Is essential for ATP hydrolysis. 1996; J Biol Chem 271: 15874–15878. 8663302 10.1074/jbc.271.27.15874

[66] Pham-Huy LA, He H, Pham-Huy C. Free radicals, antioxidants in disease and health. 2008; Int J Biomed Sci 4: 89–96. 23675073 PMC3614697

[67] Galligan JJ, Fritz KS, Backos DS, Shearn CT, Smathers RL, Jiang H, et al Oxidative stress-mediated aldehyde adduction of GRP78 in a mouse model of alcoholic liver disease: functional independence of ATPase activity and chaperone function. 2014; Free Radic Biol Med 73: 411–420. doi: 10.1016/j.freeradbiomed.2014.06.002 24924946 10.1016/j.freeradbiomed.2014.06.002 PMC4395467

[68] Carbone DL, Doorn JA, Kiebler Z, Ickes BR, Petersen DR. Modification of heat shock protein 90 by 4-hydroxynonenal in a rat model of chronic alcoholic liver disease. 2005; J Pharmacol Exp Ther 315: 8–15. doi: 10.1124/jpet.105.088088 15951401 10.1124/jpet.105.088088

[69] Galligan JJ, Smathers RL, Fritz KS, Epperson LE, Hunter LE, Petersen DR. Protein carbonylation in a murine model for early alcoholic liver disease. 2012; Chem Res Toxicol 25: 1012–1021. doi: 10.1021/tx300002q 22502949 10.1021/tx300002q PMC3423195

[70] Galligan JJ, Smathers RL, Shearn CT, Fritz KS, Backos DS, Jiang H, et al Oxidative Stress and the ER Stress Response in a Murine Model for Early-Stage Alcoholic Liver Disease. 2012; J Toxicol 2012: 207594 doi: 10.1155/2012/207594 22829816 10.1155/2012/207594 PMC3399426

[71] Smathers RL, Fritz KS, Galligan JJ, Shearn CT, Reigan P, Marks MJ, et al Characterization of 4-HNE modified L-FABP reveals alterations in structural and functional dynamics. 2012; PLoS One 7: e38459 doi: 10.1371/journal.pone.0038459 22701647 10.1371/journal.pone.0038459 PMC3368874

[72] Roede JR, Carbone DL, Doorn JA, Kirichenko OV, Reigan P, Petersen DR. In vitro and in silico characterization of peroxiredoxin 6 modified by 4-hydroxynonenal and 4-oxononenal. 2008; Chem Res Toxicol 21: 2289–2299. doi: 10.1021/tx800244u 19548352 10.1021/tx800244u

